# Phylogenetic and morphological characterization of trypanosomes from Brazilian armoured catfishes and leeches reveal high species diversity, mixed infections and a new fish trypanosome species

**DOI:** 10.1186/s13071-015-1193-7

**Published:** 2015-11-06

**Authors:** Moara Lemos, Bruno R. Fermino, Cíntia Simas-Rodrigues, Luísa Hoffmann, Rosane Silva, Erney P. Camargo, Marta M. G. Teixeira, Thaïs Souto-Padrón

**Affiliations:** Departamento de Microbiologia Geral, Instituto de Microbiologia Paulo de Góes, Centro de Ciências da Saúde, Universidade Federal do Rio de Janeiro, Bloco I, sala 019. Av. Carlos Chagas Filho, 373, Ilha do Fundão, Rio de janeiro, 21941-902 Brazil; Departamento de Parasitologia, Instituto de Ciências Biomédicas, Universidade de São Paulo, São Paulo, Brazil; Departamento de Bioquímica, Instituto de Química, Universidade de São Paulo, São Paulo, Brazil; Instituto de Biofísica Carlos Chagas Filho, Universidade Federal do Rio de Janeiro, Rio de Janeiro, Brazil; Instituto Nacional para Pesquisa Translacional em Saúde e Ambiente na Região Amazônica, Conselho Nacional de Desenvolvimento Científico e Tecnológico/MCT, Rio de Janeiro, Brazil; Instituto Nacional de Ciência e Tecnologia em Biologia Estrutural e Bioimagens, Centro de Ciências da Saúde, bloco I, Rio de Janeiro, Brazil

**Keywords:** *Trypanosoma*, Fish, Catfish, Leech, Culture, Phylogeny, Taxonomy, Morphology, Ultrastructure, Mixed infections

## Abstract

**Background:**

Several *Trypanosoma* species transmitted by leeches infect marine and freshwater fish worldwide. To date, all South American fish trypanosome species identified have been based on unreliable morphological parameters. We recently isolated and cultured trypanosomes from the Brazilian armoured catfishes *Hypostomus luetkeni* and *H. affinis*. Here, we report the first phylogenetic analyses of South American (Brazilian) trypanosomes isolated from fish, and from leeches removed from these fish. We also analysed morphologically and morphometrically the different forms of fish, leech and cultured trypanosomes.

**Methods:**

V7V8 SSU rRNA and gGAPDH sequences were used for phylogenetic analysis of Brazilian fish and leech trypanosomes. Trypanosomes from cultures, fish blood and leech samples were also characterized morphologically and morphometrically by light and electron microscopy.

**Results:**

In blood smears from fish high trypanosome prevalence (90–100 %) and parasitemia (0.9-1.0x10^2^) were observed. Phylogenetic relationships using SSU rRNA and gGAPDH showed that, despite relevant sequence divergence, all Brazilian fish (and derived cultures) and leech trypanosomes clustered together into a single clade. The Brazilian clade clustered with European, North American and African fish trypanosomes. Based on sequence analysis, we uncovered a new species of Brazilian fish trypanosome, *Trypanosoma abeli* n. sp. *Trypanosoma abeli* cultures contained pleomorphic epimastigotes, small trypomastigotes and rare sphaeromastigotes. Ultrastructural features of *T. abeli* included a cytostome-cytopharynx complex in epi- and trypomastigotes, a compact rod-like kinetoplast, lysosome-related organelles (LROs) and multivesicular bodies. Trypanosomes found in fish blood smears and leech samples were highly pleomorphic, in agreement with sequence data suggesting that catfishes and leeches often have mixed trypanosome infections.

**Conclusions:**

*Trypanosoma abeli* n. sp. is the first trypanosome from South American fishes isolated in culture, positioned in phylogenetic trees and characterized at the ultrastructural level. *Trypanosoma abeli* n. sp. is highly prevalent in *H. luetkeni* and *H. affinis* armoured catfish from the Atlantic Forest biome, and in other catfish species from the Amazon and the Pantanal. Sequencing data suggested that Brazilian catfish often have mixed trypanosome infections, highlighting the importance of molecular characterization to identify trypanosome species in fishes and leeches.

**Electronic supplementary material:**

The online version of this article (doi:10.1186/s13071-015-1193-7) contains supplementary material, which is available to authorized users.

## Background

Trypanosomes are widespread and highly prevalent in freshwater and marine fishes, including teleost and elasmobranch species. Since the initial report of trypanosomes in the blood of trouts from the species *Salmo fario* [1], more than 200 species of fish trypanosomes have been identified under the taxonomic criteria of morphology, and geographical and host origin [2, 3]. Although most infected fish are asymptomatic, fish trypanosomiasis can be severe at high parasitemias, and symptoms include anaemia, leukocytosis, hypoglycemia and splenomegaly [4, 5]. Aquatic leeches are both hosts and vectors of fish trypanosomes [6, 7]. Despite the reports of trypanosomes infecting leech-parasitizing fishes [7–10], host-vector relationships were only recently demonstrated by molecular comparison of trypanosomes from fish and leeches removed from the same fish [11].

In Brazil, more than 60 species of trypanosomes were recorded in marine and freshwater fishes [3] including loricariid (catfish) species such as *Trypanosoma hypostomi* [12], *Trypanosoma chagasi* and *Trypanosoma guaibensis* [13] and *Trypanosoma lopesi* [14]. Catfishes belong to the order Siluriformes, which comprises more than ~3,093 species of freshwater and salt water fishes inhabiting every continent except Antarctica, although more than 50 % of all catfish species, including all members of Loricariidae (>680 species), live in the tropical Americas. Loricariidae species vary largely in size, inhabit almost all freshwater habitats and are mostly bottom feeders. The genus *Hypostomus* is native to South America and comprises of large armoured catfish species used extensively as ornamental fish and food [15–17].

After the initial description of fish trypanosome cultivation by Thomson [18], several authors reported culturing of a variety of trypanosomes from the blood of freshwater and marine fish species from Europe, North America and Africa [7, 18–22]. However, only recently trypanosomes from South American (Brazilian) fish – the catfish *H. luetkeni* and *H. affinis* - were established in culture [23], despite the numerous reports of fish trypanosomes in Brazil. While there are many species descriptions based on Giemsa-stained bloodstream trypomastigote forms of fish trypanosomes [24–29], comparatively less is known about their ultrastructure, since only a few species were analysed at this level, either in culture [19, 30], or *in vivo*, in fish [5, 31, 32] and leech samples [33, 34].

Nevertheless, all descriptions of Brazilian fish trypanosome species available to date were based exclusively on morphology and host taxonomy criteria [3, 12, 14, 35–37]. These criteria are insufficient for species identification, because some species are morphologically indistinguishable (aside from intra-specific pleomorphism), and not host-specific, with a high frequency of mixed infections detected in molecular studies [11, 38–41]. Barcoding by variable SSU rRNA sequences is capable of distinguishing new trypanosome species/genotypes from previously known ones [11, 41–47].

The number of phylogenetic studies of fish trypanosomes has increased in recent years, and these studies have focused on trypanosomes removed directly from the blood of European [38, 41, 48, 49], African [11, 39] and Asian [5, 40, 50] marine and freshwater fishes. These trypanosomes segregate, in general, in two groups within a main clade [5, 11, 38, 40, 41, 49, 50]. However, molecular phylogenetic analysis currently adopted for the description of trypanosome species is still lacking for Brazilian fish trypanosomes. These limitations render necessary a molecular revision of the profusion of fish trypanosome species, including all of those described in Brazil before this study.

In this study, specimens of the ornamental armoured catfishes *H. luetkeni* and *H. affinis* were captured in The Atlantic Forest biome of Southeast Brazil and examined to assess trypanosome prevalence and parasitemia. We used phylogenetic analysis and both light and electron microscopy to characterize trypanosomes from blood samples, cultures and leeches removed from catfish. Also, the phylogenetic analysis included novel data on additional trypanosome isolates from other loricariid fishes captured in northeast (Amazonia) and central (Pantanal) Brazil, aiming to assess the genetic diversity. By integrating the phylogeny of fish trypanosomes with the morphological and ultrastructural features of cultured parasites, our findings enabled the description of *Trypanosoma abeli* n. sp. of Brazilian armoured catfishes.

## Methods

### Fish and leech collection, studied area, parasitemia and trypanosome prevalence

Armoured catfish, identified as *Hypostomus affinis* and *Hypostomus luetkeni,* were captured in the city of Guarani, state of Minas Gerais, Brazil (21°21’S, 43°02’W), in the banks of Pomba River, a 300-km long affluent of the Paraiba do Sul River basin that extends through the States of São Paulo, Minas Gerais and Rio de Janeiro, in the Atlantic Forest biome. Fish capture was performed according to procedures D-075 of the National Forests Institute (IEF) and N° 24402–1 of the Brazilian Institute of Environment and Renewable Natural Resources (IBAMA). If present, leeches were removed manually from the body surface and the oral and branchial cavities of captured fish. Leeches were macerated and sectioned in longitudinal and transversal directions for trypanosome detection. For blood sample collection, fish were anaesthetized and blood was collected by cardiac puncture, and blood from the heart, liver and kidney were used for smears. Only blood from cardiac puncture was used for trypanosome hemoculture, performed as described previously [23].

Trypanosome prevalence was determined by light microscopy examination of stained blood smears, and parasitaemia was determined by direct counting of trypanosome cells in fish blood using a hemocytometer. Samples of leeches macerated in sterile PBS were examined by light microscopy for the presence of trypanosomes. Fish blood and leech samples were processed immediately for electron microscopy (see SEM and TEM).

### Hemocultures of fish trypanosomes

Here, we used 16 cultured fish trypanosome isolates whose *in vitro* cultivation was first described by Lemos & Souto-Padrón [23]. Isolates were cultured in three different biphasic media: a) Ponsele medium overlaid with Eagle’s Basal Medium (BME) (CULTILAB, Brazil) diluted to different concentrations; b) blood agar base (BAB) (HIMEDIA, Brazil) overlaid with BME diluted to 50 %, and c) NNN blood agar base overlaid with Fish ringer’s medium. All media were supplemented with 10 % heat-inactivated fetal calf serum (FCS) and cultures were maintained at 22 °C.

### DNA extraction, PCR amplification, sequencing and phylogenetic analysis

DNA was extracted from trypanosome cultures in the exponential phase of growth using phenol-chloroform, and from fish blood and leech samples using the method described by Fermino *et al.* [44]. PCR amplification of the V7V8 region of SSU rRNA genes was performed using the primers 609 F (5’CACCCGCGGTAATTCCAGC3’) and 706R (5’TCTGAGACTGTAACCTCAA3’), and the primers GAP3F (5’GTGAAGGCGCAGCGCAAC3’) and GAP5R (5’CCGAGGATGYCCTTCATG3’) were used for the amplification of gGAPDH sequences. The PCR reaction conditions employed for the two sequences were detailed previously [45]. Amplified DNA fragments were cloned in pGEM-T-easy, and 3–5 clones were sequenced for each sample, except for culture L4100, for which an additional 15 clones were sequenced, aiming at detecting mixed trypanosome infections. Sequences were deposited in GenBank under the accession numbers shown in Table 1. For comparative purposes, DNA from blood trypanosomes of loricariid fishes captured in the Amazonia and the Pantanal biomes (Fermino *et al*., in preparation) were included in this study (Table 1).

**Table 1:** Host, geographic origin, and SSU rRNA and gGAPDH sequences of fish and leech trypanosome isolates

Isolate	Host	City/Coordenates	SSU rRNA	gGAPDH	Organism
Cultures					
H27100^a^	*H. affinis*	Rio Pomba-MG/21°27’S 43°18’W	KR052821		*T. abeli*
L450	*H. luetkeni*	Rio Pomba-MG/21°27’S 43°18’W	KR048309		*T. abeli*
L460	*H. luetkeni*	Rio Pomba-MG/21°27’S 43°18’W	KR048306		*T.* sp*.*
L4100	*H. luetkeni*	Rio Pomba-MG/21°27’S 43°18’W	KR048310	KR048292	*T. abeli*
L6NBA	*H. luetkeni*	Rio Pomba-MG/21°27’S 43°18’W	KR048307		*T. abeli*
L7fish^a^	*H. luetkeni*	Rio Pomba-MG/21°27’S 43°18’W	KR048308		*T. abeli*
P130	*H. affinis*	Rio Pomba-MG/21°27’S 43°18’W		KR048293	*T. abeli*
P1100	*H. affinis*	Rio Pomba-MG/21°27’S 43°18’W		KR048294	*T. abeli*
P250	*H. affinis*	Rio Pomba-MG/21°27’S 43°18’W		KR048295	*T. abeli*
P350	*H. affinis*	Rio Pomba-MG/21°27’S 43°18’W		KR048296	*T. abeli*
P3100	*H. affinis*	Rio Pomba-MG/21°27’S 43°18’W		KR048297	*T. abeli*
P450	*H. affinis*	Rio Pomba-MG/21°27’S 43°18’W		KR048298	*T. abeli*
P4100	*H. affinis*	Rio Pomba-MG/21°27’S 43°18’W		KR048299	*T. abeli*
P560	*H. affinis*	Rio Pomba-MG/21°27’S 43°18’W		KR048300	*T. abeli*
P5100	*H. affinis*	Rio Pomba-MG/21°27’S 43°18’W		KR048301	*T. abeli*
PO8R	*H. luetkeni*	Rio Pomba-MG/21°27’S 43°18’W		KR048302	*T. abeli*
Blood					
BSC100	*L. anisitsi*	Miranda-MS/19°57’S 57°011 W	KR048304	KR048287	*T*. sp.
CRCPE03	*H. affinis*	C. do Rio Claro-MG/20°98’S 46°11’W	KR048305		*T*. sp.
H27^a^ ^b^	*H. affinis*	Rio Pomba-MG/21°27’S 43°18’W		KR048291	*T*. sp.
L7^a^ ^b^	*H. luetkeni*	Rio Pomba-MG/21°27’S 43°18’W		KR048290	*T*. sp.
BSC451	*Ancistrus* sp.	Amazon Basin		KR048303	*T. abeli*
Leech					
SSH2^b^	*Haementeria* sp	Rio Pomba-MG/21°27’S 43°18’W	KR052820		*T*. sp.
TSC11	*Haementeria* sp	Miranda-MS/19°57’S 57°011 W		KR048288	*T*. sp.
TSC13	*Haementeria* sp	Miranda-MS/19°57’S 57°011 W		KR048289	*T*. sp.

^a^Sequences obtained from cultures and blood samples

^b^mixed infections

Trypanosome SSU rRNA and gGAPDH gene sequences determined in this study were aligned with those from several fish trypanosomes available in GenBank. Two alignments were created: One comprising SSU rRNA sequences (800 ~ bp of variable V7V8 region of SSU rRNA) from Brazilian fish trypanosomes in cultures and trypanosomes from leeches, aligned with sequences from freshwater and marine fish trypanosomes from Europe, Africa and Asia; trypanosomes from crocodilians, lizards and snakes were used as outgroup. The second alignment was composed of gGAPDH sequences (~600 bp) of fish trypanosomes obtained from cultures, blood samples and leeches taken from captured fishes. This alignment includes GenBank sequences from a variety of ‘Aquatic clade’ trypanosomes (found in fishes and in other aquatic and semi-aquatic vertebrates), as well as those from trypanosomes outside the Aquatic clade, and other non-trypanosome trypanosomatids, which were used as outgroups. The complete list of samples, host and geographic origin, and Genbank accession numbers of all sequences determined in this work are shown in Table 1. Phylogenetic analysis of the new sequences (barcodes) were done by maximum parsimony using V7V8 SSU rRNA sequences (dendrogram of Aquatic clade trypanosomes), and maximum likelihood (ML), maximum parsimony (MP) and Bayesian inference (BI) for inferences of phylogenetic trees based on gGAPDH sequences, as described previously [43, 44, 46, 47].

### Light microscopy and morphometry

Smears of fish blood samples, leech gut contents and cultures were fixed with methanol, stained with Giemsa, and examined using a Zeiss Axioplan II light microscope equipped with a Color View XS digital camera. Morphometry analysis of the different developmental forms was performed using the AnalySIS® soft, using images of 80 bloodstream trypomastigotes (20 images per developmental form). Data were analysed by descriptive statistics and Kruskal-Wallis variance analysis (*p* < 0.05 was considered statistically significant, using the BioEstat software, version 5.0).

### Fluorescence microscopy

Trypanosomes in the logarithmic phase of growth were washed in BME at 4 °C and adhered to glass coverslips coated with 0.1 % of poly-l-lysine in PBS (pH 7.2), for 20 min. Then, cells were washed, fixed in methanol at 20 °C, and incubated at 22 °C with 1 μg/mL Hoechst H33342 (Molecular Probes) in BME, for 15 min. Coverslips were mounted using 0.2 M N-propyl-gallate (Sigma, USA) in glycerol and 0.01 M PBS (pH 7.2), and analyzed by epifluorescence microscopy using a Zeiss Axioplan II light microscope equipped with a Color View XS digital camera.

### Scanning Electron Microscopy (SEM) and Transmission Electron Microscopy (TEM)

Blood and axenic trypanosome cultures were fixed in 2.5 % glutaraldehyde in 0.1-M cacodylate buffer (pH 7.2) containing 5 mM calcium chloride and 3.7 % sucrose. For SEM, cells were washed and adhered to glass coverslips previously coated with 0.1 % of poly-l-lysine and processed (dehydrated, critical point dried and coated), according Lemos *et al.* [51]. Leeches were sectioned in the longitudinal and transverse directions, fixed and critical point dried for SEM (as described above), adhered to glass coverslips using adhesive tape, and coated with a 20-nm-thick gold layer, using a Bal-Tec CPD030 sputtering device. SEM samples were examined in a QUANTA 250 (Fei Company) Scanning Electron Microscope operated at 15 kV.

For TEM, flagellates from blood samples, cultures and leeches were fixed as described above and post-fixed for 30 min (in the dark) in 1 % osmium tetroxide (OsO_4_), 0.8 % potassium ferrocyanide and 5 mM calcium chloride, in 0.1 M cacodylate buffer (pH 7.3). Then, samples were washed, dehydrated in a series of acetone solutions of ascending concentrations, and embedded in Polybed 812 resin. Ultrathin sections were stained with uranyl acetate and lead citrate, and observed in a FEI Morgagni F268 Transmission Electron Microscope (Eindhoven, The Netherlands), operating at 80 kV.

## Results and discussion

### Prevalence of trypanosomes in armoured catfishes and in leeches removed from these fishes

In this study, Giemsa-stained blood smears from 40 specimens of *H. affinis* were all positive for trypanosomes by microscopy, yielding 100 % prevalence, with an average of 0.9 × 10^2^ parasites/ml. The prevalence of trypanosomes in 10 specimens of *H. luetkeni* was 90 %, and the average parasitaemia was 1 × 10^2^ parasites/ml. Similar levels of parasitaemia were detected in blood samples collected from the heart, liver and kidneys, for both fish species (Additional file 1). The fish trypanosome *T. cobitis* concentrates in the visceral circulation, mainly of the kidney of the hosts *Cottus gobio, Phoxinus phoxinus, Nemacheilus barbatulus* and *Gobio gobio* [9], while *T. mioxocephali* concentrates in the heart of *Myoxocephalus octodecimspinosus* [52]. These studies suggested that blood samples obtained from the heart and kidneys may allow improved sensitivity for the detection of fish trypanosomes, since these may be a preferred infection site for some trypanosomes.

The first reports of fish trypanosomes in Brazil date from the early 20th century, and surveys performed in several regions described diverse trypanosomes in catfish [35, 53, 54]. Splendore [12] was the first to provide data on the prevalence (9.5 %) of *Trypanosoma hypostomi* in blood smears from *Hypostomus aurogutatus* caught in the Tietê River (SP)*.* Bara and Serra-Freire [13] reported high prevalences of *Trypanosoma chagasi* (95 %) and low of *Trypanosoma guaibensis* (7 %) in *Hypostomus punctatus.* Several armoured fish species captured in the hydrological basin of the Guamá River (in the state of Pará, in Northern Brazil) showed variable prevalence of trypanosome infection; the prevalence in *Hypostomus* sp. was of 20 %, and these fish showed the highest parasitaemia and haematological changes, but no evidence of disease [55]. Here, despite relevant parasitaemia, no macro pathological signs were observed in *H. affinis* and *H. luetkeni* infected with trypanosomes.

The leeches removed from *H. affinis* and *H. luetkeni* were identified morphologically as *Haementeria* sp. and molecularly as *Haementeria brasiliensis* [56]. On average, two and nine leeches per fish (for *H. affinis* and *H. luetkeni*, respectively) were found attached to the skin, mouth and, occasionally, to the pectoral fins of armoured catfish, at all seasons. The prevalence of trypanosomes in leeches was 81 % and 75 %, for leeches taken from *H. affinis* and *H. luetkeni,* respectively*.* The number of trypanosomes infecting leeches was high, ranging from 1.6 to 1.1 × 10^4^ parasites/ml of leech macerate. Our data showing high trypanosome prevalence in leeches infecting fish is in line with that reported in previous studies: 61 % of *Actinobdella inequiannulata* leeches were infected by *T. catostomi* [57] and 60–100 % of *Johanssonia arctica* leeches were infected with *T. murmanensis* [58]. Importantly, these leech species were capable of transmitting trypanosomes to fish, by feeding [10]. High prevalence was also reported in the leech *Z. arugamensis*, considered the vector of *Trypanosoma nudigobii* [11]. In Brazil, a single report related the presence of trypanosomes in freshwater leeches (from the species *Batracobdella gemmata*) removed from *H. punctatus* catfish [59]; however, no prevalence data was reported in that study*.*

### Culture of fish trypanosomes

The isolation and *in vitro* maintenance of fish trypanosomes has been a challenge since the initial description of *T. danilewskyi* isolation [18]*.* Recently, we isolated trypanosomes from Brazilian fish for the first time, successfully culturing these parasites in haemocultures [23]. In the present study we analysed 16 of these culture isolates obtained from 6 *H. affinis* and 4 *H. luetkeni* specimens*.*

### Barcoding of Brazilian fish and leech trypanosomes and phylogenetic relationships

Most molecular and phylogenetic studies of fish trypanosomes relied on SSU rRNA sequences, and show a major clade harbouring all fish trypanosomes, divided in two clades of species from marine or freshwater fishes. The positioning of trypanosomes from turtles and platypuses was unresolved, but they always clustered closely related to fish trypanosomes in the phylogenetic trees [5, 11, 38–41, 50].

**Fig. 1:**
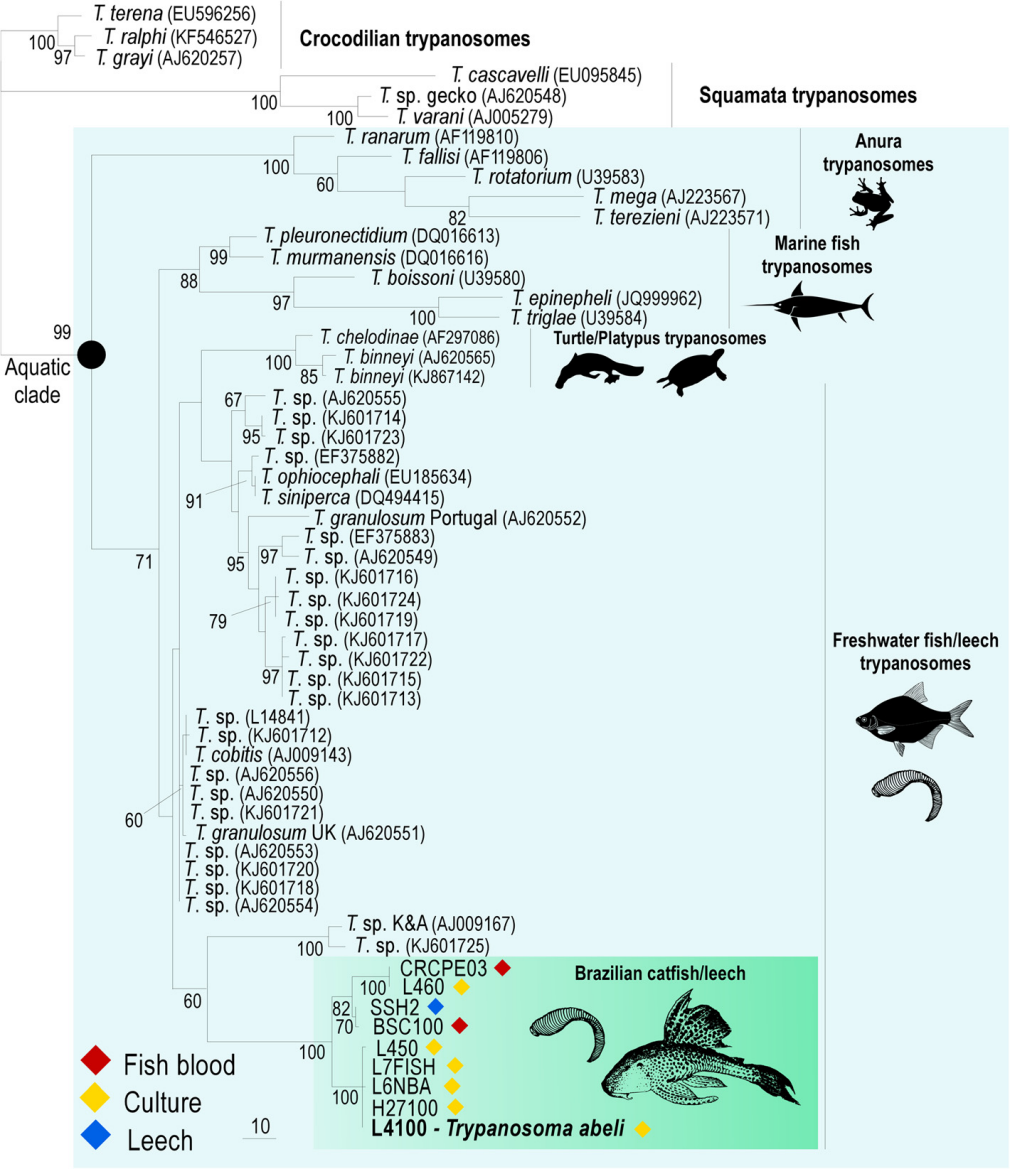
Barcoding and phylogenetic relationships of fish trypanosomes. Phylogenetic analysis inferred by maximum parsimony based on V7V8 SSU rRNA sequences (barcodes) of fish trypanosomes from Brazilian armoured catfish (Loricariidae), or from leeches taken from these fishes. Note the inclusion of a new species, *Trypanosoma abeli* n. sp. established in culture. Freshwater and marine fish trypanosomes from different continents were included in the analysis, as well as other trypanosome species of the Aquatic clade. Numbers at nodes are bootstrap values derived from 100 replicates. GenBank accession numbers of SSU rRNA sequences determined in this study are listed in Table 1. Codes in parenthesis are GenBank accession numbers available for previously described species

In the present study, PCR-amplified trypanosome SSU rRNA and gGAPDH sequences obtained directly from fish blood samples had to be cloned before sequencing due to the presence of more than one sequence per sample, indicating that the Brazilian armoured catfishes examined in the present study had mixed trypanosome infections (Table 1). Although other trypanosome species were identified by sequence analyses, the lack at this moment of appropriate type-materials prevented their description as additional species. According to the V7V8 SSU rRNA analysis, sequences from Brazilian fish and leech trypanosomes examined in this study clustered together in a clade with small heterogeneity (average of ~0.8 %); however, relevant sequence divergences (up to 3.8 %) separated this clade from available sequences from European, Asian and African fish trypanosomes (Fig. 1). The closest relatives of the Brazilian fish trypanosome was *T*. sp. K&A [60] from the European freshwater leech *Piscicola geometra*, and a trypanosome from European percae (*Perca fluviatilis*) [41] (Fig. 1). Sequences of trypanosomes from diverse fish orders and families, including catfishes from other parts of the world, did not cluster with those from Brazilian loricariid catfish, or from leeches that were attached to these fish (Fig. 1). Other catfish trypanosomes included in the phylogenetic analysis were isolated from widespread species of Clariidae (Africa), Siluridae (Europa) and Bagridae (Asia) [38, 39, 41].

**Fig. 2:**
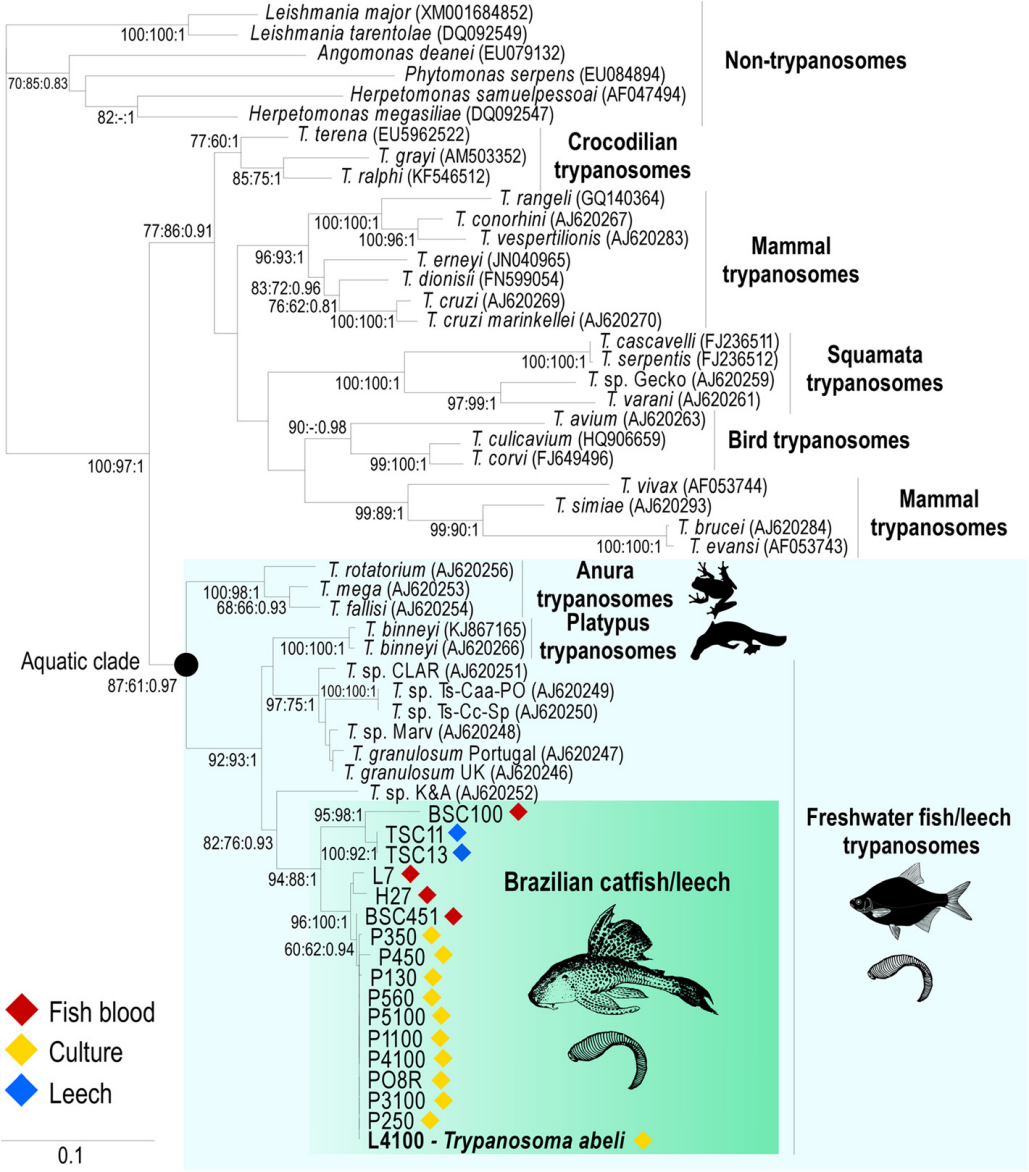
Phylogenetic tree of fish trypanosomes with the positioning of *Trypanosoma abeli* n. sp.: Maximum likelihood phylogenetic tree inferred from gGAPDH sequences of *Trypanosoma abeli* (cultures) and other trypanosomes from Brazilian loricariids and leeches, including sequences from European, African and Asian fish trypanosomes, as well as turtle and platypus trypanosomes placed within the Aquatic clade. Sequences from other trypanosomes and non-trypanosome trypanosomatids were used as outgroups in the phylogenetic tree (− Ln = − 7078.905197). Numbers on branches represent bootstrap support (>50) estimated with 500 pseudoreplicates in RAxML, using GTRGAMMA. GenBank accession numbers of gGAPDH sequences determined in this study are listed in Table 1. Codes in parenthesis are GenBank accession numbers available for previously described species

Aside from yielding highly consistent alignments, gGAPDH sequences are much more polymorphic than SSU rRNA sequences for most trypanosome clades, resulting in phylogenies with considerably improved resolution [43, 44, 46, 47, 61]. Indeed, our analysis showed an average of 3.8 % gGAPDH sequence divergence within the clade formed by the Brazilian trypanosomes of fishes and leeches, compared with ~0.8 % of divergence for V7V8 SSU rRNA sequences. The phylogenetic inferences based on gGAPDH strongly supported the positioning of the trypanosomes found in Brazilian *Hypostomus* catfishes, as well as of the whole assemblage of fish trypanosomes (Fig. 2).

To better assess the genetic diversity, we compared gGAPDH sequences obtained from blood samples and cultures of trypanosomes from Brazilian catfishes and leeches removed from these fishes. We detected sequence polymorphisms among different blood samples from the same fish specimen (not shown), but also between samples from fishes and their respective leeches, and between samples from different specimens of the same fish species. Importantly, blood samples frequently harboured different sequences, indicating mixed trypanosome infections, in contrast to cultured isolates, from which, in general, only one sequence was recovered, likely due to selection by culturing (Table 1). It appears likely that more mixed samples would be identified by sequencing a larger number of cloned PCR-amplified DNA sequences obtained from blood samples of each fish specimen.

All trypanosome gGAPDH sequences from Brazilian armoured catfish obtained in this study clustered together in phylogenetic inferences (Fig. 2), supporting the existence of a separate clade exclusive for trypanosomes from Brazilian catfishes and from leeches taken from these fishes. The clade of Brazilian catfish trypanosomes was nested into the “Aquatic clade” composed by trypanosomes from anurans, fishes, turtles, platypuses and aquatic leeches [38, 61–63]. In agreement with previous phylogenetic studies [38, 39, 41], all fish trypanosomes nested into a single clade that also harboured trypanosomes from turtles and platypuses; this complex branching pattern needs the inclusion of more trypanosomes for improved resolution.

In our study, no sequence from trypanosomes found in leeches completely matched those found in the corresponding fish blood samples. In contrast, a recent study reported identical SSU rRNA sequences for trypanosomes in marine fishes and leeches taken from the same fishes, confirming host-vector relationships [11]. Our findings support the existence of a large repertoire of trypanosome species and genotypes infecting Brazilian catfishes and leeches, including relevant divergences distinguishing trypanosomes found in sympatric *Hypostomus* fishes and within the same fish specimens. Therefore, results from the present study reinforce the need for molecular characterization using sensitive approaches in order to evaluate species repertoire before ascertaining any parasite-vector association between trypanosome species infecting these hosts.

Taken together, our phylogenetic analyses using V7V8 SSU rRNA and gGAPDH sequences support the identification of a clade of new trypanosome species from Brazilian loricariid fishes differing from previously molecularly characterized fish trypanosomes, including those from other catfish species (Figs. 1 and 2). This clade may be associated with the evolutionary history of the Loricariidae restricted to Central and South America [15, 16]. In contrast, phylogenetic studies of fish trypanosomes from Europe, Africa and Asia did not support geographical structure or host-restrictions at species, genus or family levels [11, 38, 41]. Further studies are required to verify whether trypanosomes from other Neotropical fish families also cluster within this clade.

### Description of *Trypanosoma abeli* n. sp. in Brazilian armoured catfishes

Fish trypanosomes characterized in this study shared similar sequences regardless of host species (*H. affinis* and *H. luetkeni)*. Within the group of sequences obtained, we identified unique SSU rRNA and gGAPDH sequences (as determined by BLAST searches and positioning in phylogenetic trees) that supported the description of a new trypanosome species – hereby named *Trypanosoma abeli* n. sp. (Figs. 1 and 2). Here, we characterized this species using as type material the cultured isolate L4100, which showed a single trypanosome sequence in the analysis of sequences from 15 clones of the PCR-amplified DNA. In addition to *H. affinis* and *H. luetkeni* captured in Southeast Brazil, in the Atlantic Forest biome, isolates of *T. abeli* n. sp. sharing identical or very similar sequences were found in loricariids from other genera captured in Amazonia (*Ancistrus* sp.) and The Pantanal (*Liposarcus anisistis*) biomes (Figs. 1 and 2).

In the absence of cultures, new fish trypanosome species have been described based on the combination of morphological and molecular phylogenetic data from blood flagellates [5, 11, 40, 50]. Our finding that fish trypanosomes often harbour mixed infections, combined with similar data from previous studies [40, 41], suggests that this approach requires a careful assessment to exclude the possibility of mixed infections, which preclude the unambiguous association between trypanosome sequences and parasite morphotypes found in fish blood and leech samples.

### Morphological and developmental analyses of *Trypanosoma abeli* n. sp. in cultures

**Fig. 3:**
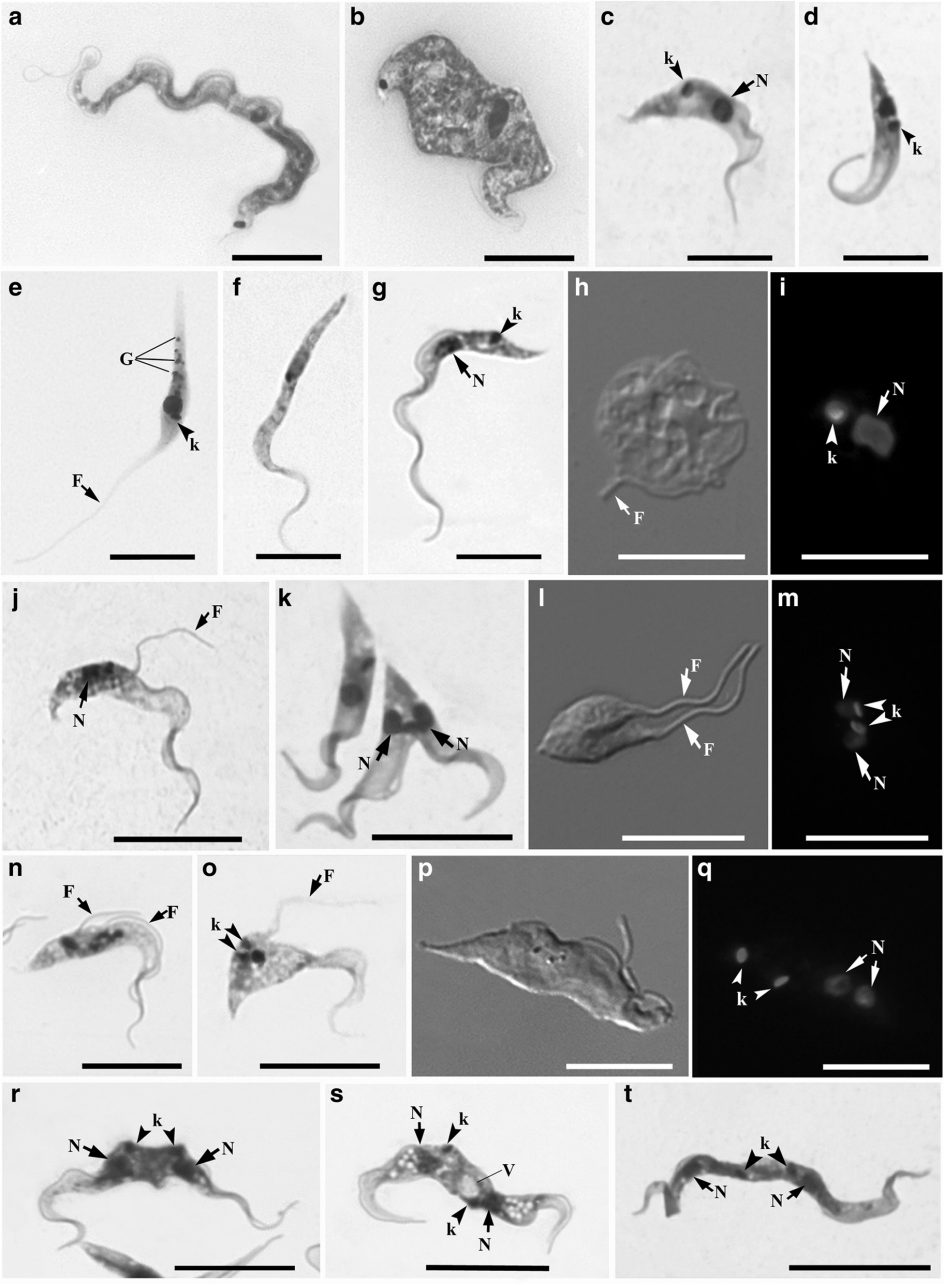
Light microscopy analysis of *Trypanosoma abeli* n. sp. cultured *in vitro*. Parasites were visualized by Giemsa staining (**a**-**g**, **j**, **k**, **n**, **o**, **r**-**t**), differential interference contrast (**h**, **l**, and **p**), and fluorescence microscopy using Hoechst H33342 (**i**, **m**, and **q**). Elongated (**a**) and short and broad (**b**) bloodstream trypomastigotes. Short trypomastigotes (**c**) and ‘pyriform’ (i.e. “pear shaped”) epimastigotes (**d** and **e**) and were the first developmental forms observed in primary cultures. Although trypomastigotes (**g**) were again observed in stationary phase, as well as spheromastigotes (**h** and **i**), with a round kinetoplast (arrowhead in **i**) and short flagellum (arrow in **h**). Cultures contained dividing epimastigotes (**j**-**m**) and trypomastigotes (**n**-**t**) at different stages of cell division, with duplicated single-copy structures, including the flagellum (F), the nucleus (N) and the kinetoplast (k). G, granules; V, vacuoles. Scale bar, 10 μm

In primary hemocultures, bloodstream trypomastigotes (Fig. 3, a and b) transformed into short trypomastigotes (Fig. 3c) and, within two days, into epimastigotes (Fig. 3d). Established cultures in the logarithmic phase of growth consisted mostly of epimastigotes that ranged in shape and size from small and often “pear-shaped” (Fig. 3 d and e) to elongated and slender (Fig. 3f), with a long flagellum, a rod-like kinetoplast and an oval nucleus. Small (Fig. 3c) and elongated (Fig. 3g) trypomastigotes, as well as spheromastigotes with a round kinetoplast and a short free flagellum (Fig. 3, h and i), appeared in stationary-phase cultures. We also observed dividing epimastigotes (Fig. 3, j-m) and trypomastigotes (Fig. 3, n-t). In dividing trypomastigotes that had not reached the stage of cytokinesis, the two kinetoplasts located considerably posterior to the nuclei (Fig. 3, p and q). In cells that seemed to be at later stages in cell division, the kinetoplasts and nuclei appeared closer to each other (Fig. 3 r and s), but probably separated once again later in cytokinesis (Fig. 3t).

Measurements of culture forms (type material) are still important for species description and must be included in the taxonomic section. Thus, we performed a detailed morphometric analysis of the different cell types found in cultures of isolate L4100 of *T. abeli* n. sp. (Additional file 2). Slender and pyriform epimastigotes had 19.8 ± 6.3 and 13.3 ± 5.4 μm in body length along cell midline, respectively, while trypomastigotes were longer, with body lengths along cell midline of 34.4 ± 1.6 and 22.8 ± 5 μm, for long and short forms, respectively.

### Scanning electron microscopy (SEM) of fish and leech trypanosomes

**Fig. 4:**
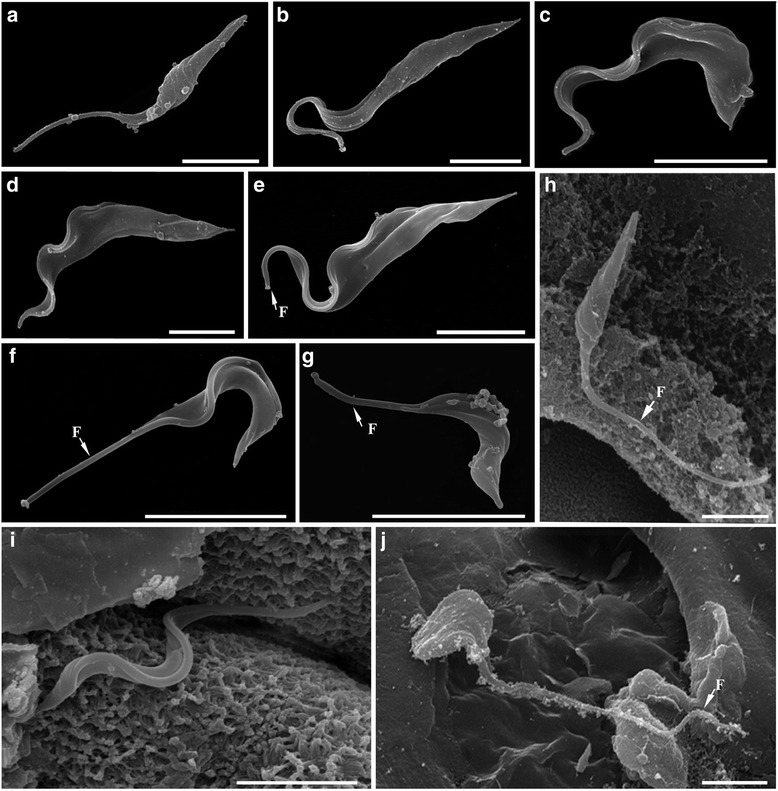
Morphological analysis of Brazilian catfish trypanosomes by scanning electron microscopy (SEM). **a**-**d** *Trypanosoma abeli* n. sp. **e**-**g** Bloodstream trypomastigotes of *Trypanosoma* sp. from fish blood samples. **h**-**j** *Trypanosoma* sp. from leech samples. Cultured epimastigotes with pyriform (**a**) or elongated (**b**) bodies. Short (**c**) and elongated (**d**) cultured trypomastigotes of *T. abeli*. Bloodstream trypomastigotes had elongated (**e**) or short (**f** and **g**) bodies, with a long section of ‘free’ flagellum portion (arrows). Samples from leech stomach caecum contained pyriform epimastigotes (**h**), short trypomastigotes (**i**) and short and wide forms (**j**) similar to spheromastigotes. Scale bars, 5 μm

The SEM analysis of *T. abeli* n. sp. highlighted the morphological diversity of cultured epimastigotes (Fig. 4, a and b) and trypomastigotes (Fig. 4, c and d), corroborating the light microscopy findings. The pleomorphism of bloodstream trypanosomes from *H. affinis* and *H. luetkeni* was clear in SEM images (Fig. 4 e-g). Trypanosomes from fish blood and leeches were, in fact, too pleomorphic (possibly due, in part, to mixed infections) to allow identification of the forms that corresponded to the bloodstream trypomastigotes of *T. abeli*. Flagellates found in the stomach caeca of leeches (Fig. 4h-j) were similar to those observed in cultures of *T. abeli*, and comprised of epimastigote (Fig. 4h), elongated trypomastigote (Fig. 4i) and spheromastigote (Fig. 4j) forms.

### Ultrastructural analysis of *Trypanosoma abeli* n. sp. by transmission electron microscopy (TEM)

**Fig. 5:**
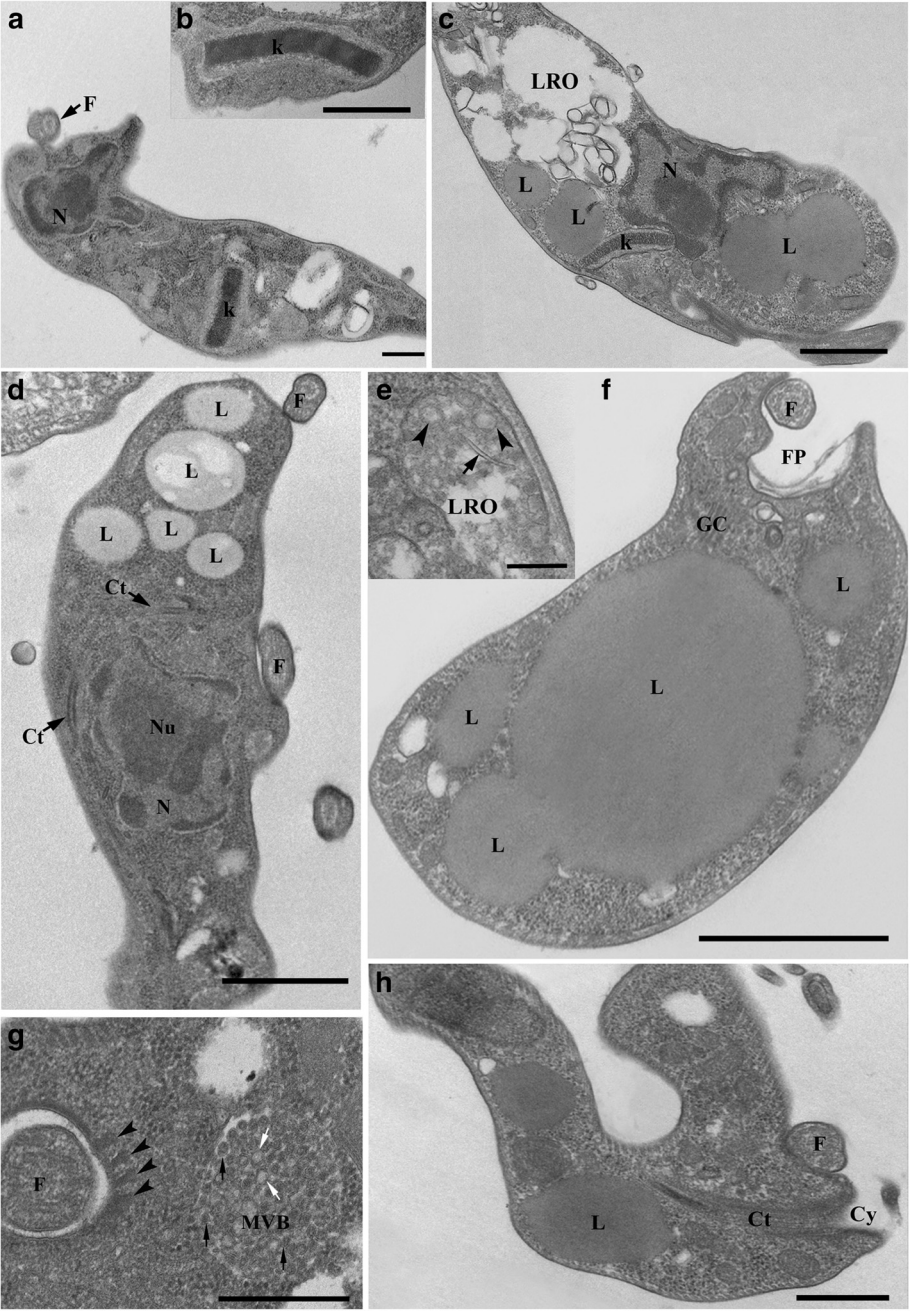
Morphological analysis of culture forms *Trypanosoma abeli* n. sp. by transmission electron microscopy (TEM). Cultured trypomastigotes (**a**) had a rod-like kinetoplast (k) similar to that of bloodstream trypomastigotes of *Trypanosoma* sp. from *H. affinis* and *H. luetkeni* (**b**), and epimastigotes of *T. abeli* (**c**). Lysosome related organelles (LROs; in **c** and **e**) had an electron-lucent matrix containing small vesicles (arrowheads in **e**) and rod-shaped electron-lucent bodies (arrows in **e**). Epimastigotes (**c**) and trypomastigotes (**d**) had numerous lipid bodies (L) of different sizes located near the kinetoplast (k) and the nucleus (N). Adjacent lipid bodies appeared to fuse, generating larger ones (**f**). A multivesicular body (MVB) containing small electron lucent (white arrow) and electron dense (black arrows) vesicles was located near the flagellar pocket (**g**), and a microtubule quartet (arrowheads in **g**) subtended the flagellar pocket membrane. Trypomastigotes had a cytostome (Ct)-cytopharynx (Cy) complex (**d** and **h**) that extended deep into the cytoplasm (**h**), reaching the perinuclear region (**d**). Nucleus, N; Nu, nucleolus; k, kinetoplast; FP, flagellar pocket; F, flagellum, Ac, acidocalcisomes; GC, Golgi Complex; L, lipid body; LRO, lysosome-related organelle; MVB, multivesicular body; Cy, cytostome; Ct, cytopharynx (Ct). Scale bars: 1 μm (**c, f**); 500 nm (**a, b, h**); 250 nm (**d**); 200 nm (**e**); 400 nm (**g**)

To examine in detail the ultrastructure of *T. abeli* n. sp., we analysed log-phase cultures of this parasite by transmission electron microscopy (TEM). When observed by TEM, *T. abeli* n. sp. trypomastigotes and epimastigotes had a rod-like kinetoplast containing a compact array of kDNA fibrils (Fig. 5, a and c) similar to that observed in the kinetoplast of bloodstream trypomastigotes found in *H. affinis* and *H. luetkeni* (Fig. 5 b). The kinetoplast pattern observed in *T. abeli* was similar to that of freshwater and marine fish trypanosomes, such as *T. pseudobagre* and *T. triglae* [31, 32], but differed from that of *T. cruzi*, which exhibits a round kinetoplast with a relatively ‘loose’ kDNA network [64].

Numerous lipid bodies with variable sizes (from 50 nm to 1.5 μm) were observed in the cytoplasm of *T. abeli* n. sp. (Fig. 5c, d, f), and we also observed smaller lipid bodies that appeared to fuse with (or bud from) larger ones (Fig. 5f). At the posterior region, *T. abeli* n. sp. had large membrane-bound structures reminiscent of lysosomes-related organelles (LROs), containing linear membrane profiles and small vesicles (Fig. 5e) immersed in an electron-dense matrix (Fig. 5c). These putative LROs were morphologically similar to those of *T. cruzi* (referred to as ‘reservosomes’) [65, 66], which contain rod-shaped electron-lucent bodies [67] also observed in *T. abeli* n. sp. (Fig. 5e).

Epimastigotes of *T. abeli* n. sp. had multivesicular bodies near the flagellar pocket containing numerous vesicles of regular size (~80 nm) and with an electron-dense matrix (Fig. 5g). Multivesicular bodies were also observed in *Trypanosoma terena* from cayman [44], *Trypanosoma serpentis* and *Trypanosoma cascaveli* from snakes [43] and *Trypanosoma cobitis* from fish [34]; however, they had different localization and internal vesicles of different size compared with the *T. abeli* n. sp. multivesicular bodies. The nature of these structures in trypanosomatids remains unknown, and hypotheses suggest that they are formed by viral particles, or may be associated with the endocytic or exocytic pathways [34, 68].

Both epimastigotes and bloodstream trypomastigotes of *T. abeli* n. sp. displayed structures morphologically similar to acidocalcisomes, an acidic organelle rich calcium and polyphosphates and involved in osmoregulation, in some protozoa [69]. These structures have been described in other fish trypanosomes (*Trypanosoma pseudobagri* and *T. danilewskyi*) [31, 32], and in *Trypanosoma fallisi* from anurans [70], *T. serpentis* and *T. cascavelli* from snakes [43], *T. terena* and *T. ralphi* from caymans [44], as well as in several trypanosomes of mammals, including *T. cruzi, Trypanosoma erneyi* and *Trypanosoma brucei* [46, 71].

The cytostome-cytopharynx complex - an invagination of plasma membrane penetrating deep into the cytoplasm and sometimes reaching the perinuclear region - was observed in trypomastigotes (Fig. 5d, h) and epimastigotes (not shown) of *T. abeli*. In some images, the cytostome appears to ‘open’ into the flagellar pocket (not shown), differently from that observed in other trypanosomes [30, 31, 34, 43, 44, 47, 64, 70, 72]. The cytopharynx was first observed in epimastigotes and trypomastigotes of the fish trypanosomes *T. raiae, T. danilewskyi* and *T. cobitis* [30, 31, 34], and a cytostome-cytopharynx complex was also observed in epimastigotes of trypanosomes from anurans, snakes and crocodilians [43, 44, 70]. The cytostome-cytopharynx complex has also been observed in epimastigotes of *T. cruzi* and in other species of the subgenus *Schyzotrypanum*; however, trypomastigotes of these species lacked this complex [46, 72, 73]. Thus far, the presence of the cytostome-cytopharynx complex in blood and culture trypomastigotes appears to be a common and exclusive characteristic of fish trypanosomes [31], as confirmed here by our TEM analysis of *T. abeli* n. sp.

### Morphology features of blood trypomastigotes found in *H. affinis* and *H. luetkeni*

**Fig. 6:**
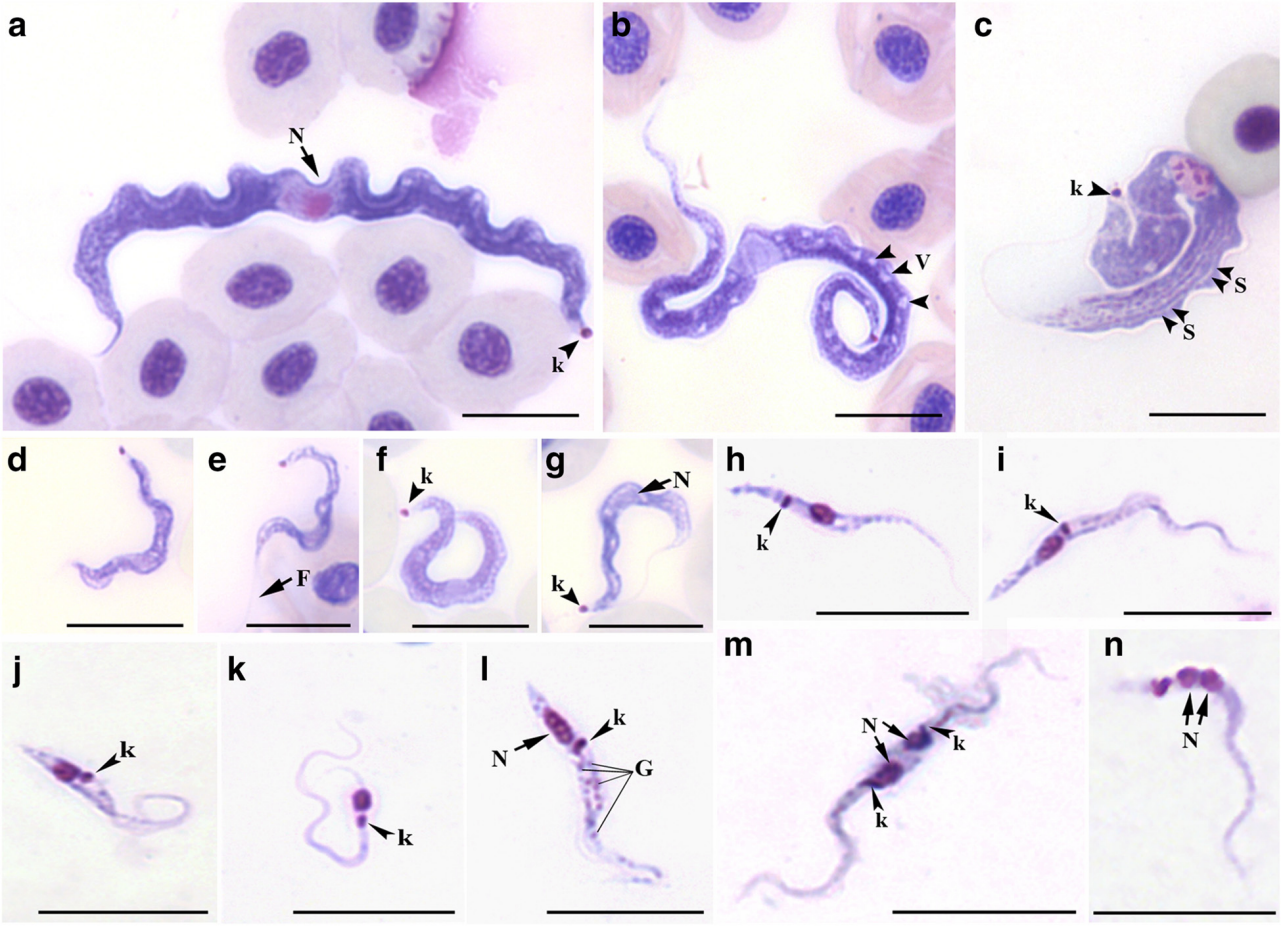
Morphological analysis of different developmental forms of *Trypanosoma* sp. observed in the blood of *H. affinis* and *H. luetkeni* (**a-g**), or in the gut contents of *Haementeria brasiliensis* leeches removed from these fish (**h**-**n**)*.* Bloodstream trypomastigotes were divided into four morphotypes as follows: **a** morphotype 1 has a long and broad body; **b** morphotype 2 has a long and slender body, and several vacuoles (arrowheads); **c** morphotype 3 has a broader body with striations (arrowheads); and **d-g** morphotype 4 has a short and slender body. The developmental forms isolated from leeches were trypomastigotes (**h**), slender epimastigotes (**i, k,** and **l**), and pyriform epimastigotes (**j**). Dividing forms included epimastigotes undergoing cytokinesis (**m**), with two nuclei (arrows) and 2 kinetoplasts (arrowheads), and trypomastigotes at a post-mitotic stage of the cell duplication cycle (N; arrows indicate duplicated nuclei). N, nuclei; F, flagellum; k, kinetoplast; S, striae; V, vacuoles; G, granules. Scale bar = 10 μm

Bloodstream trypomastigotes from both *H. affinis* and *H. luetkeni* were highly polymorphic (Fig. 6), and the number and proportion of morphotypes varied between blood samples. The morphological variation observed here, if used as the sole taxonomic criterion, would support the description of more than one species in these samples. In fact, the different morphotypes may represent distinct developmental stages from the same species, instead of different species. In fish blood samples, we could not distinguish between intra- and inter-specific polymorphism and, thus, could not assign a given morphotype to a given species.

To facilitate morphological description of blood flagellates, morphometrical data were used to divide bloodstream trypomastigotes into four morphotypes, according to their body length along cell midline (short vs. elongated, with *p* < 0.001 by Kruskal-Wallis) and width (slender vs. broad, *p* < 0.00001 by Kruskal-Wallis) (Fig. 6, a-g; Additional file 3). Morphotype 1 trypomastigotes (Fig. 6a; Additional file 3) have a long and broad body (67.3 ± 1.2 μm in length and 4.08 ± 0.1 μm in width), with a tapering anterior end, a broad posterior end (often C-shaped), and a long section of free flagellum (20.8 ± 0.6 μm). The nucleus is elongated and positioned in the anterior end, and the kinetoplast is close to the posterior extremity of the cell. Morphotype 2 (Fig. 6b; Additional file 3) has a long and slender body (64.57 ± 2.3 μm length and 2.73 ± 0.06 μm width) and a long free flagellum (21.07 ± 1.4 μm). The rod-like kinetoplast is close to the posterior end, the nucleus is oval and eccentrically placed in an anterior position, and the cytoplasm may contain several vacuoles. Morphotype 3 (Fig. 6c; Additional file 3) has a long, broad and S-shaped body (length 61 ± 3 μm, width 5.5 ± 0.4 μm), with striations along the main axis, a tapered anterior end, a wide posterior end, and a long (41.6 ± 8.8 μm) free flagellum. A round kinetoplast is found at the posterior end and the nucleus is almost oval, large and anterior in position (Fig. 6c). Morphotype 4 (Fig. 6, d-g; Additional file 3) has a short and slender body (length 28.01 ± 1.4 μm, width 1.58 ± 0.08 μm), a short free flagellum (13.49 ± 1.4 μm), a rod-like kinetoplast located at the posterior end, and an anterior and oval-shaped nucleus (Fig. 6 d-g), similar to that observed in morphotypes 2 and 3.The four morphotypes described here may represent a mixture of trypanosome species/genotypes as indicated by our sequence analyses (Figs. 1 and 2; Table 1)*.*

There are more than 30 trypanosome species reported in Brazilian catfishes and at least 25 of armoured catfishes [3], including ~15 species described in *Hypostomus* spp. However, no trypanosome species was described in *H. affinis* or *H. luetkeni* before this study*.* The morphotypes identified in the blood samples of catfishes examined in this study broadly resembled those previously reported in other catfishes. However, the trypomastigotes observed in blood smears from fishes infected with *T. abeli* n. sp. were in general longer and larger than those found in other species of armoured catfishes, although trypomastigotes of different trypanosome species share morphological similarities with at least one of the four morphotypes identified in fishes infected with *T. abeli*. For instance, the trypomastigotes of *Trypanosoma chagasi* are quite similar to morphotype 1 [55], whereas *Trypanosoma loricariae* [35] and *Trypanosoma hypostomi* [12] share blood forms more similar to morphotype 2 of *T. abeli*. Altogether, our findings corroborated that morphology and morphometry are not sufficient to distinguish species of fish trypanosomes, although these data are relevant as a complementary data for the description of any new trypanosome species.

### Morphological features of trypanosomes found in leeches removed from *H. affinis* and *H. luetkeni*

Developmental forms of trypanosomes were observed in the digestive tract of *H. brasiliensis* leeches taken from *H. affinis* and *H. luetkeni*. Trypomastigotes (Fig. 6h) were observed in the proboscis and stomach caeca, and epimastigotes (Fig. 6, i-l) in the stomach caeca and intestines. Slender (Fig. 6 i, k and l) and pyriform (Fig. 6j) epimastigotes had an oval nucleus and a rod-like kinetoplast. Trypomastigotes had slender bodies, with an oval nucleus located in the anterior region of the cell body, and a rod-like kinetoplast close to the nucleus (Fig. 6h). Leech samples also contained dividing epimastigote (Fig. 6, m) and trypomastigote (Fig. 6n) forms, including trypomastigotes with two nuclei (Fig. 6n). Morphometrical data of trypomastigotes and epimastigotes observed in leech samples are shown in Additional file 4.

In leeches examined in a previous study, spheromastigotes occurred in the crop, and this and a variety of epimastigote forms were observed in the intestine, while metacyclic trypomastigotes were generally found in the proboscis following complete digestion of the blood meal. However, migration to the leech proboscis sheath by fish trypanosomes has not been elucidated [7, 8, 11, 57].

Variable forms of trypanosomes in leeches can represent mixtures of trypanosome species, different stages of one trypanosome species, or flagellates from old and new infections [11]. Our results show that leeches recovered from fish could be infected with trypanosomes different from those infecting their host fish. Unfortunately, we did not recover *T. abeli* n. sp. sequences from DNA preparations from *Haementeria brasiliensis* leeches. Therefore, trypanosomes found in Giemsa-stained leeches samples could be not identified as *T. abeli* n. sp., and further surveys are necessary to evaluate the role of *H. brasiliensis* as vector of this trypanosome species*.*

### Taxonomical summary

Phylum Euglenozoa Cavalier-Smith, 1981. Class Kinetoplastea Honigberg, 1963, emend. Vickerman, 1976. Order Trypanosomatida Hollande 1952. Family Trypanosomatidae Doflein, 1951 *Trypanosoma abeli* n. sp. Lemos and Souto-Padrón 2015.

#### Type material

Hapantotype, culture of the isolate L4100 from *Hypostomus luetkeni* deposited in the FIOCRUZ Protozoan Collection (code COLPROT - 700) of the Instituto Oswaldo Cruz, Fundação Oswaldo Cruz, Rio de Janeiro, RJ, Brazil*.* Details of *T. abeli* has been registered in ZooBank with the following Life Science Identifier (LSID): zoobank.org:pub:AAD60F84-2E99-46A4-929B-BBA1AEDE437C.

#### Type host

*Hypostomus luetkeni* (Siluriformes, Loricariidae). Additional host: *Hypostomus affinis* (Siluriformes, Loricariidae).

#### Type locality

Pomba River (21°21’S, 43°02’W), a small tributary of the Paraíba do Sul River, Minas Gerais, Southeast Brazil.

#### Morphological characteristics

In logarithmic cultures, pyriform (length, 13.3 μm; width, 1.6 μm) and slender (length, 19.8 μm; width, 1.7 μm) epimastigotes (Fig. 3 e-f). In stationary cultures, elongated (length, 34.4 μm; width, 1.6 μm; Fig. 3 c) and short (length, 22.8 μm; width, 2.1 μm) trypomastigotes (Fig. 3g). Ultrastructural features include a rod-shaped and compact kinetoplast, LRO structures, multivesicular bodies and a cytostome-cytopharynx complex (Fig. 5a, c, e, g and h).

#### Species diagnosis

Unique sequences of SSU rRNA and gGAPDH of *T. abeli* n. sp.*,* deposited in GenBank under accession numbers KR048310 (SSU rRNA gene) and KR048292 (gGAPDH gene).

#### Etymology

The species was named in honour of José Abel, for his constant encouragement and genuine interest in science.

## Conclusions

*Trypanosoma abeli* n. sp. is the first trypanosome from South American fishes isolated in culture, positioned in phylogenetic trees, and characterized at morphological and ultrastructural levels. Phylogenetic relationships inferred using SSU rRNA and gGAPDH gene sequences positioned the trypanosomes of armoured catfishes within an assemblage of closely related trypanosomes from Brazilian loricarids and leeches removed from these fishes, but separated from the other fish trypanosomes, all from other continents. Our studies revealed the presence of *T. abeli* n. sp. in *H. luetkeni* and *H. affinis* caught in a small river of the Atlantic Forest, and in other loricariid fishes from rivers of Amazonia and the Pantanal biomes.

Ultrastructural analyses confirmed that *T. abeli* n. sp. have a cytostome-cytopharynx complex in both epimastigotes and trypomastigotes, as shown in other fish trypanosomes, large lysosome-related organelles and multivesicular bodies. In spite of considerable resemblance between blood trypomastigotes morphotypes detected in blood smears of *H. luetkeni* and *H. affinis*, we obtained molecular evidence for mixed trypanosome infections in these fishes, which prevented the establishment of morphotype-species associations. In addition, we obtained different sequences from fish blood samples and from leeches taken from these fishes; thus, further and more detailed molecular characterization is required to assess the repertoire of *H. luetkeni* and *H. affinis* trypanosomes and any link between these trypanosomes and *H. brasiliensis* leeches.

## Additional files

Additional file 1:
**Trypanosome parasitemia in blood removed from organs of** ***Hypostomus affinis*** **and** ***H. luetkeni*** **armored catfish.** (XLSX 8 kb) Additional file 2:
**Morphometric analysis of the different** ***in vitro*** **cultured forms of** ***Trypanosoma abeli*** **n. sp.** (XLSX 9 kb) Additional file 3:
**Morphometric analysis of bloodstream trypomastigotes.** (XLSX 10 kb) Additional file 4:
**Morphometric analysis of the different developmental forms of trypanosomes found in leeches of the species** ***Haementeria brasiliensis*** **.** (XLSX 9 kb)
